# Integrating Molecular Markers and Environmental Covariates To Interpret Genotype by Environment Interaction in Rice (*Oryza sativa* L.) Grown in Subtropical Areas

**DOI:** 10.1534/g3.119.400064

**Published:** 2019-03-15

**Authors:** Eliana Monteverde, Lucía Gutierrez, Pedro Blanco, Fernando Pérez de Vida, Juan E. Rosas, Victoria Bonnecarrère, Gastón Quero, Susan McCouch

**Affiliations:** * Plant Breeding and Genetics Section, School of Integrative Plant Science, Cornell University, Ithaca NY 14853; † Department of Agronomy, University of Wisconsin - Madison WI 53706; ‡ Programa Nacional de Investigación en arroz, Instituto Nacional de Investigación Agropecuaria (INIA), INIA Treinta y Tres 33000, Uruguay; § Unidad de Biotecnología, Instituto Nacional de Investigación Agropecuaria (INIA), Estación Experimental Wilson Ferreira Aldunate 90200, Uruguay; ** Department of Plant Biology, College of Agriculture, Universidad de la República, Montevideo, Uruguay

**Keywords:** rice, genotype-by-environment interaction, genomic prediction, QTL by environment interaction, environmental covariates

## Abstract

Understanding the genetic and environmental basis of genotype × environment interaction (G×E) is of fundamental importance in plant breeding. If we consider G×E in the context of genotype × year interactions (G×Y), predicting which lines will have stable and superior performance across years is an important challenge for breeders. A better understanding of the factors that contribute to the overall grain yield and quality of rice (*Oryza sativa* L.) will lay the foundation for developing new breeding and selection strategies for combining high quality, with high yield. In this study, we used molecular marker data and environmental covariates (EC) simultaneously to predict rice yield, milling quality traits and plant height in untested environments (years), using both reaction norm models and partial least squares (PLS), in two rice breeding populations (*indica* and *tropical japonica*). We also sought to explain G×E by differential quantitative trait loci (QTL) expression in relation to EC. Our results showed that PLS models trained with both molecular markers and EC gave better prediction accuracies than reaction norm models when predicting future years. We also detected milling quality QTL that showed a differential expression conditional on humidity and solar radiation, providing insight for the main environmental factors affecting milling quality in subtropical and temperate rice growing areas.

Genetic by environment interaction (G×E) could be expressed as a difference in the relative response of genotypes across diverse environments. When we consider a set of genotypes exposed to different environments, their performance will differ depending on the interaction of genetic properties with the different environmental conditions, leading to differences in variances and rank changes among genotypes (Cooper and DeLacy 1994). These rank changes represent a very important challenge for breeders due to the difficulties of selecting genotypes with stable performance over diverse environments.

Environments can be different both in time and space. For this reason, the concept of G×E embraces both interactions that take place between genotypes and a particular location (genotype by location interaction), and between genotypes and particular years (genotype by year interaction). Genotype by location interactions are usually determined by soil and climate conditions, while genotype by year interactions are characterized by plot-to-plot variability and weather conditions (Malosetti *et al.* 2016).

Several statistical approaches have been proposed to describe G×E in the context of classical plant breeding. The classic parametric approaches used to evaluate G×E are based on linear regression and ANOVA techniques. Linear regression analysis (Yates and Cochran 1938; Finlay and Wilkinson 1963) measures individual genotype performance over environmental means. Multiplicative models combine univariate and multivariate approaches for reducing data dimensionality and facilitate the interpretation of results (Gauch 1988; Smith *et al.* 2005). The most commonly used multiplicative model is the additive main effect and multiplicative interaction model (AMMI; Gauch 1992). AMMI combines univariate (ANOVA) and multivariate (singular value decomposition; SVD) techniques for estimating genotype and environment main effects, and G×E effects, respectively. Factorial regression models are another type of models that allow the modeling of genotype sensitivity to specific environmental covariates (EC) (van Eeuwijk *et al.* 1996; Vargas *et al.* 1998; Malosetti *et al.* 2004; Malvar *et al.* 2005). Linear mixed-models became very popular for the analysis of G×E since they allow different correlation structures among environments (Burgueño *et al.* 2007). These covariance structures may range from a compound symmetry form, where homogeneous variance and homogeneous covariance between environments are assumed, to an unstructured form where a covariance parameter is assumed between each pair of environments and environments are assumed to have heterogeneous variances.

Recent developments in sequencing technologies and statistical modeling have made it possible to use dense genotypic information to predict phenotypic responses through genomic prediction (GP). This idea was introduced by Meuwissen *et al.* (2001), and provides an alternative approach to indirect selection in crop breeding. GP models were originally developed for traits evaluated in single environments, but more recently standard GP models have been extended to account for G×E. Burgueño *et al.* (2012) were the first to extend genomic best linear unbiased prediction (GBLUP) to a multi-environment context, by combining genetic and environmental covariance matrices and using different covariance structures to model the environmental component. Lopez Cruz *et al.* (2015) proposed a marker by environment approach where marker effects and genotypic values are partitioned into main effects across environments (stability) effects that are specific to each environment (interactions).

Standard GP models can be modified to accommodate climate information in the form of EC. However, including EC in the analysis can pose some similar constraints encountered when predicting breeding values with multiple markers. As climatic and agronomic systems develop, a very high number of covariates can potentially be obtained increasing the dimensionality of the data and also the possibility of being correlated with each other. Several studies have proposed different ways to deal with highly dimensional data, showing that the incorporation of explicit environmental and genetic information can improve prediction accuracies and predict performance in untested environments (Heslot *et al.* 2014; Jarquín *et al.* 2014; Malosetti *et al.* 2016). Jarquín *et al.* (2014) proposed a Bayesian reaction norm model where the main genetic and environmental effects were modeled using covariate structures as functions of molecular markers and EC respectively, and the interaction effects between markers and EC were modeled using a multiplicative operator. Heslot *et al.* (2014) proposed a factorial regression model, where instead of using all the available EC and molecular markers, they chose the EC that most significantly influenced the growth and development of the crop by using crop growth models (CGM). These variables were introduced in the factorial regression model along with those markers that showed the most variable effects across environments and reducing thus, dimensionality of both markers and EC.

The partial least square regression (PLS) (Wold *et al.* 2001) is a generalization of multiple linear regression (MLR). PLS is a dimension reduction approach that can accommodate a large number of correlated genetic and environmental variables simultaneously, by finding one or few factors named latent variables (LV) that explain both the variance of the X matrix (containing predictor variables) and the covariance between matrices X and Y (containing response variables). PLS can be used for variable selection, in order to improve estimation/prediction performance, but also to improve model interpretation and understanding of the system studied. Another advantage of PLS is that it can be more robust against multicollinearity (Aastveit and Martens 1986). PLS models have previously been used for GP both in plant and animal breeding (Solberg *et al.* 2009; Long *et al.* 2011; Colombani *et al.* 2012; Iwata *et al.* 2015), to detect highly influential environmental and marker covariates that explain a significant proportion of the total G×E (Vargas *et al.* 1998; Crossa *et al.* 1999; Vargas *et al.* 1999).

Understanding the genetic basis of G×E is also necessary to gain predictive capability, and one way to do this is detecting QTL with varying effects across different environmental conditions, or QTL by environment interaction (QTL×E). Methods usually employed to detect QTL×E have been very useful to detect QTL with differential expression across environments, but provide no explanation of the underlying environmental factors involved. When weather data are available, factorial regression models can be used to determine the extent of influence of these factors on QTL×E (Crossa *et al.* 1999; Campbell *et al.* 2004; Malosetti *et al.* 2004).

Rice is one of the world’s most important staple food crops, constituting over 21% of the caloric intake of the world’s population and up to 76% of the caloric needs in many Asian countries (Fitzgerald *et al.* 2009). World markets dictate the value of rice mainly based on milling quality traits, so breeding for both high yield and quality is a major breeding objective for rice exporting countries like Uruguay.

In a previous study we showed that accounting for heterogeneous covariance parameters between pairs of environments can be beneficial for predicting yield and milling quality performance in Uruguayan rice for untested environments (Monteverde *et al.* 2018). In another study, Quero *et al.* (2018) found a set of QTL for milling yield traits in the same Uruguayan *indica* and *tropical japonica* populations. However, none of these studies tested the use of EC to both predict yield and milling quality traits in untested environments, and investigate QTL responses in specific environments. The main objectives of this study were to: 1) use molecular marker data and environmental covariates simultaneously to predict rice yield and milling quality traits in untested environments (years), and 2) Detect marker by environment covariate interactions that provide explanations of variable QTL effects across environments. Two rice breeding populations (*indica* and *tropical japonica*) were used in this study and were evaluated for grain yield, plant height and grain quality traits (head rice percentage and chalky grain percentage) across 3-5 years in Eastern Uruguay. Results from these two analyses provided clues about the main environmental variables that could be driving G×E in temperate rice-growing regions such as Uruguay.

## Materials and Methods

### Germplasm

The germplasm consists of two rice-breeding populations, an *indica* and a *tropical japonica* population belonging to the National Institute of Agricultural Research (INIA-Uruguay). Both populations were evaluated in a single location, Paso de la Laguna Experimental Station (UEPL), Treinta y Tres, Uruguay (33°15’S, 54°25’W) between 2009-2013.

The *indica* population consisted of 327 elite breeding lines evaluated over three years (2010-2012), and the field design consisted in a randomized complete block design with two or three replications. Trait correlations, heritabilities, and genomic prediction accuracies for this dataset were computed in previous studies (Rosas *et al.* 2017, Monteverde *et al.* 2018, Quero *et al.* 2018). The *tropical japonica* population consisted of 320 elite breeding lines evaluated over five years (2009-2013). The number of accessions observed each year ranged from 93 to 319, as detailed in Table 2. This dataset was unbalanced with non-random missing data, since ∼50% genotypes were dropped from testing every year based on performance, and new genotypes were added over time. Each year, the genotypes were planted independently in replicated trials in six-row plots using an augmented randomized complete block design with two or three replications. Both *indica* and *tropical japonica* trials were conducted under irrigated conditions using appropriate pest and weed control.

The agronomic traits of interest used in this study were Grain Yield (GY of paddy rice in kilograms per hectare) and Plant Height (PH measured in cm from the soil surface to the tip of the flag leaf). The grain quality traits measured were Percentage of Head Rice Recovery (PHR measured in grams, as the weight of whole milled kernels, using a 100g sample of rough rice), and the percentage of Chalky Grain (GC measured as % of chalky kernels in a subsample of 50 g of total milled rice, where the area of chalk -core, white back or white belly- was larger than half the kernel area based on visual inspection). More details about how grain quality traits were measured can be found in Quero *et al.* (2018) and Monteverde *et al.* (2018).

### Phenotypic analysis

Phenotypic data for each trait were analyzed separately each year. The model used to calculate the best linear unbiased estimators (BLUEs) for each year was:

$$y_{ijkl}=\mu +b_{i}+g_{j}+r_{k(i)}+c_{l(i)}+\varepsilon_{ijkl}$$

where $y_{ijkl}$ is the trait score, μ is the overall mean, $b_{i}$ is the random effect of the i^th^ block with $b_{i}∼N\left(0,\sigma_{b}^{2}\right)$, where $\sigma_{b}^{2}$ is the block variance, $g_{j}$ is the genotypic effect of the j^th^ genotype, $r_{k(i)}$ and $c_{l(i)}$ are the random k^th^ row and l^th^ column effects nested within the i^th^ block with $r_{k(i)}∼N\left(0,\sigma_{r}^{2}\right)$ and $c_{l(i)}∼N\left(0,\sigma_{c}^{2}\right)$, where $\sigma_{r}^{2}$ and $\sigma_{c}^{2}$ are the row and column variances respectively, and $\varepsilon_{ijkl}\;$is the model residual vector with $\varepsilon_{ijkl}∼N\left(0,\sigma_{\varepsilon }^{2}\right)$ where $\sigma_{\varepsilon }^{2}$ is the error variance.

Trait heritabilities in *tropical japonica* for years 2009 and 2013 (data not yet published) was calculated on a per line basis as $H^{2}=\sigma_{g}^{2}/\left(\sigma_{g}^{2}+\sigma_{\varepsilon }^{2}/r\right)$, where $\sigma_{g}^{2}$ is the variance among genotypes, $\sigma_{\varepsilon }^{2}$ is the error variance, and *r* is the number of replicates.

### Genotypic characterization

The lines were genotyped using genotyping-by-sequencing (GBS). SNP calling was performed using the TASSEL 3.0 GBS pipeline (Bradbury *et al.* 2007), and SNPs were aligned to the Nipponbare reference genome MSU version 7.0 (http://rice.plantbiology.msu.edu/) using Bowtie 2 (Langmead and Salzberg 2012). Imputation of missing data were performed with the FILLIN algorithm implemented in TASSEL 5.0 (Swarts *et al.* 2014) for both datasets separately. The GBS datasets were filtered to retain markers with <50% missing data after imputation, and a minor allele frequency MAF > 0.05, as reported by Quero *et al.* (2018), and Monteverde *et al.* (2018). The final *indica* and *tropical japonica* marker dataset consisted of 92,430 and 44,598 SNP markers respectively.

### Derivation of EC From weather data

Daily weather data were obtained from GRAS unit from INIA (http://www.inia.uy/gras/Clima/Banco-datos-agroclimatico). The database contains weather data from 1965 to the last calendar month completed, for all 5 INIA experimental stations in Uruguay. The variables available were related to temperature, precipitation, solar radiation, humidity, wind, and evaporation.

To compute the EC from daily weather data for each rice genotype, the plant development stage has to be determined in order to account for the differential effect that weather variables may have in different stages of crop development. This information is usually hard to obtain directly or, as in our case, not available. For this reason, the phenology of the crop was defined according to flowering time (days to 50% flowering, FT), which was measured for each line every year. With this measure, and sowing and harvest date, three main phenology stages were determined for each year, according to Yoshida (1981): vegetative stage (of variable length, starting on sowing date), reproductive stage (starting 35 days before FT), and maturation stage (ending 30 days after FT).

Once these phenological stages are defined for each year, EC can be computed from daily weather data. Covariates with zero variance were removed from the analysis. For prediction, both markers and EC were centered by subtracting the mean, and standardized to unit variance by dividing the centered values by the standard deviation of the marker or EC. A total of 54 EC were used in both populations (18 for each developmental stage: vegetative, reproductive, and maturation), and are summarized in Table 1.

**Table 1:** Environmental covariates used in this study

EC abbreviation	Explanation
ThermAmp	Thermal Amplitude (°C): Average of daily thermal amplitude calculated as max temperature (°C) – min temperature (°C).
RelSun	Relative sunshine duration (%): Quotient between the real duration of the brightness of the sun and the possible geographical or topographic duration.
SolRad	Solar radiation (cal/cm2/day): Solar radiation calculated using the Armstrong’s formula.
EfPpit	Effective Precipitation (mm): Average of daily precipitation in mm that is actually added and stored in the soil.
DegDay	Degrees Day in rice (°C): Mean of Daily average temperature minus 10 °.
RelH	Relative humidity (hs): Sum of daily amount of hours (0hs-24hs) where the relative humidity was equal to 100%.
PpitDay	Precipitation day: Sum of days when it rained.
MeanTemp	Mean Temperature (°C): Average of temperature over 24 hs (0-24 hs).
AvTemp	Average Temperature (°C): Average Temperature calculated as daily (Max+Min)/2.
MaxTemp	Maximum Temperature (°C): Average of maximum daily temperature.
MinTemp	Minimum Temperature (°C): Average of minimum daily temperature.
TankEv	Tank water evaporation (mm): Amount of evaporated water under influence of sun and wind.
Wind	Wind speed (2m/km/24hs): Distance covered by wind (in km) over 2m height in one day.
PicheEv	Piche Evaporation (mm): Amount of evaporated water without the influence of the sun.
MinRelH	Minimum relative humidity (%): Lowest value of relative humidity for the day.
AccumPpit	Accumulated precipitation (mm): Daily accumulated precipitation.
Sunhs	Sunshine duration: Sum of total hours of sunshine per day
MinT15	Minimum temperature below 15°: Sum of the days where the minimum temperature was below 15°.

### PLS regression

PLS regression was first introduced by Wold (1966), and was originally developed for econometrics and chemometrics. It is a multivariate statistical technique that was designed to deal with the p>>n problem; *i.e.*, when the number of explanatory variables (p) is much larger (and more highly correlated) than the number of observations (n). A brief explanation of PLS relating one response variable (y) to a set of explanatory variables (X) is given below, but it can be extended to more than one response variable (Boulesteix and Strimmer 2006; Wold *et al.* 2001).

In PLS, the data for p explanatory variables are given by the matrix $X=\left(x_{1},\ldots ,x_{p}\right)$, and data for the dependent variables are given by the response vector y. Each $x_{1},\ldots ,x_{p}$ and y vectors have n×1 dimensions corresponding to the number of observations. In this work, the y vector contains all the observations for a given trait in different environments (years), and the columns of the X matrix are the variables corresponding to either markers only, or markers and EC. All variables in PLS must be centered and scaled.

PLS is based on the latent variable (LV) decomposition:

$$X=TP^{T}+E,$$ (1)

$$y=Tq^{T}+f,$$ (2)

where T is a n×c matrix giving the LV (also called scores) for the n observations, and P (p×c) is a matrix of p-dimensional orthogonal vectors called X-loadings, q (1×c) is a vector of scalars and, also named Y-loadings, E_(n×p)_ and f_(n×1)_ are a residual matrix and vector respectively.

The LV matrix T that relates the X matrix to the vector y is calculated as:

T=XW, (3)

where W is a (p×c) matrix of weights. For a given matrix W, the LV obtained by forming corresponding linear transformations of the variables in X, $X_{1},\ldots ,X_{p}$ are denoted as $T_{1},\ldots ,T_{c}$:

$$\begin{array}{c} T_{1}=w_{11}X_{1}+\ldots +w_{p1}X_{p}\\ \vdots \\ T_{c}=w_{1c}X_{1}+\ldots +w_{pc}X_{p} \end{array}$$

These LV are then used for prediction in place of the original variables. After computing the T matrix, $q^{T}$ is obtained as the least squares solution of Equation (2):

$$q^{T}={\left(T^{T}T\right)}^{-1}T^{T}y.$$

The vector b of regression coefficients for the model y=Xb+f, to predict new responses, is calculated as:

$$b=Wq^{T}=W{\left(T^{T}T\right)}^{-1}T^{T}y.$$

Since regression and dimension reduction are performed simultaneously, all b, T, W, P and q are part of the output. Both X and y are taken into account when calculating the LV in T. Moreover, they are defined so that the covariance between the LV and the response is maximized.

In PLS, the optimal number of LV (c) must be determined. In this work, we used the root means squared error of prediction (RMSEP),

$$RMSEP=\sqrt{\frac{1}{10}\overset{10}{\underset{k=1}{\sum }}\left({\hat{y}}_{k}-y_{k}\right)}$$

which was minimized with 10-fold cross-validation in the training data set and for each value of LV (Mevik and Cederkvist 2004). In this study, two PLS models were fitted: the PLS-G model used marker covariates as predictors, and the PLS-GW model, which used both marker covariates and EC as predictors. PLS models calculations were performed with the R package “mixOmics” (Lê Cao *et al.* 2016).

### Genomic Best Linear Prediction (GBLUP) and reaction norm models

Mixed linear models were used as a baseline comparison of prediction accuracies with PLS models. The models used considered the random main effects of markers (G model), the random main effects of markers and EC (G+W model), and the random main effects of markers, EC, and the interactions between them (G+W+GW model).

The G model constituted of a standard GBLUP model for the mean performance of genotypes within each set of environments, using the following model:

$$y_{i}=\mu +g_{i}+\varepsilon_{i},$$ (4)

where μ is the overall mean, $g_{i}$ is the genotypic random effect of the i^th^ line expressed as a regression on marker covariates of the form: $g_{i}=\overset{p}{\underset{m=1}{\sum }}x_{im}b_{m}$, where $x_{im}$ is the genotype of the i^th^ line at the m^th^ marker, and $b_{m}$ is the effect of the m^th^ marker. Marker effects are considered as IID draws from normal distributions of the form $b_{m}∼N\left(0,\sigma_{b}^{2}\right)$.

The vector g=Xb contains the genomic values of all the lines, and follows a multivariate normal density with null mean and covariance matrix $Cov\left(g\right)=G\sigma_{g}^{2}$, where G is a genomic relationship matrix whose entries are given by $G=XX^{T}/p$_._

As previously reported by Jarquín *et al.* (2014), it is possible to model the environmental effects with a random regression on the EC that describes the environmental conditions faced by each genotype, that is: $w_{ij}=\overset{Q}{\underset{q=1}{\sum }}W_{ijq}\gamma_{q}$, where $W_{ijq}$ is the value of the q^th^ EC evaluated in the ij^th^ environment × genotype combination, $\gamma_{q}$ is the main effect of the corresponding EC, and Q is the total number of EC. Again, we consider the effects of the EC as IID draws from normal densities, $\gamma_{q}∼N\left(0,\sigma_{\gamma }^{2}\right)$. The vector w=Wγ follows a multivariate normal density with null mean and a covariance matrix proportional to Ω whose entries are computed the same way as those of the G matrix but using EC instead of markers. This covariance structure describes the similarity among environmental conditions. Then, the model becomes:

$$y_{ij}=\mu +w_{ij}+g_{j}+\varepsilon_{ij}$$ (5)

This model also includes a marker × EC interaction term, where the covariance of the interaction is modeled by the Hadamard product of $Z_{g}GZ_{g}^{T}$ and Ω, denoted as $\left[Z_{g}GZ_{g}^{T}\right]\circ \Omega$, where $Z_{g}$ is an incidence matrix for the vector of additive genetic effects. This model extends Equation (4) as follows:

$$y_{ij}=\mu +w_{ij}+g_{j}+gw_{ij}+\varepsilon_{ij},$$ (6)

with $w∼N\left(0,\Omega \sigma_{w}^{2}\right)$, $g∼N\left(0,G\sigma_{g}^{2}\right)$, $gw∼N\left(0,\left[Z_{g}GZ_{g}^{T}\right]\circ \Omega \sigma_{gw}^{2}\right)$, $\varepsilon ∼N\left(0,\sigma_{\varepsilon }^{2}\right)$.

### Assessing prediction accuracy for new environments

The prediction problem studied here was that of predicting future seasons, also denoted as “leave one environment out” prediction scenario. This prediction was performed by including phenotypic records and parameter information of either two (*indica* dataset) or four (*tropical japonica* dataset) years in the training population to predict a third (*indica* dataset) or fifth (*tropical japonica* dataset) year, where no phenotypic data were collected. Prediction accuracies obtained from both PLS and Reaction norm models were assessed by calculating the Pearson correlation between the predicted values from each model for a particular testing year, and the observed phenotypic values for that same year.

### QTL by EC interactions

For the detection of QTL by environment interaction we used a two-step strategy as described in Gutiérrez *et al.* (2015). In the first step, we scanned the genome of both *indica* and *tropical japonica* subspecies to detect QTL in individual environments (single environment QTL mapping). In the second step, QTL expression across environments was regressed on environmental covariates in order to explain QTL effects in terms of sensitivities to environmental covariates (Malosetti *et al.* 2004; Boer *et al.* 2007; Malosetti *et al.* 2013).

For the first step, we fitted a mixed model for single environment QTL detection. The model used was the kinship model with:

y =Xβ+Zu+e,

where y is the vector of phenotypic means for that environment, X is the molecular marker score matrix, β is the vector of marker effects, Z is an incidence matrix, u is the vector of random background polygenic effects with variance $\sigma_{u}^{2}=K\sigma_{G}^{2}$ (where K is the kinship matrix, and $\sigma_{G}^{2}$ is the genetic variance), and e is the vector of residuals. A GWAS analysis for each dataset, trait and environment was performed using the R statistical software (R Core Team 2017) with the package GWASpoly (Rosyara *et al.* 2016) fitting the additive model. For QTL determination in each environment, we used the Benjamini-Hochberg FDR (α=0.05) to control the type I error (Benjamini and Hochberg 1995).

In the second step, all marker-trait associations detected in the first step were fitted in a second mixed model testing for interaction with all available EC. This model assumes a linear relationship between the effect of the QTL and a given environmental covariate, using the model presented in Malosetti *et al.* (2013) given by:

$$y_{ij}=\mu +E_{j}+x_{i}\left(\alpha_{q}+\beta_{q}z_{j}+{\underset{¯}{a}}_{jq}\right)+{\underset{¯}{G}}_{i}+{\underline{GE}}_{ij}$$

where $y_{ij}$ is the phenotype of individual i at environment j, μ is the general mean, $E_{j}$ effect of the j^th^ environment, $x_{i}$ is the value of the i^th^ marker predictor, $\alpha_{q}$ is the effect of the q^th^ QTL in the average environment, $\beta_{q}$ corresponds to the change of the QTL effect per unit of change of the covariable’s value, and ${\underline{a}}_{iq}$ is the random effect corresponding to the residual (unexplained) QTL effect, with ${\underline{a}}_{iq}∼N\left(0,\sigma_{aq}^{2}\right)$, ${\underset{¯}{G}}_{i}$ is the random remaining (not due to the QTL) genotype effect with ${\underset{¯}{G}}_{i}∼N\left(0,K\sigma_{G}^{2}\right)$, and ${\underline{GE}}_{ij}$ is the remaining (random) G×E effect, with ${\underline{GE}}_{ij}∼N(0,\Sigma )$. All EC were tested for interaction using three different models for the variance-covariance matrix **Σ** were compared: compound symmetry (CS) where the genetic variances are homogeneous across environments $\left(\sigma_{G}^{2}+\sigma_{GE}^{2}\right)$ and the genetic covariances between environments are modeled by $\sigma_{G}$; heterogeneous compound symmetry (HCS), which allows for heterogeneous genetic variances across environments ($\sigma_{G_{j}}^{2}$) and a common genetic covariance parameter $\sigma_{G}^{2}$; and the unstructured (UN) model with a specific genetic variance parameter per environment and a specific genetic covariance between environment. The different models were compared using the Bayesian information criterion (BIC) to select the optimal model (Broman and Speed 2002). We tested for the significance of the fixed terms in mixed models using Wald test at a *p* value of 0.05, following Malosetti *et al.* (2004). For QTLxEC interaction testing we used the Benjamini-Hochberg FDR (α=0.05) to control the type I error (Benjamini and Hochberg 1995).

Mixed models for QTL×EC interaction were computed with the R package *sommer* (Covarrubias-Pazaran 2016).

### Data Availability

All the data used in this study, as well as Supplemental Table 1 are provided in Supplemental Material available at Figshare. Genotype data can be found as RDS files (“geno_indica.rds” and “geno_japonica.rds”), phenotypic data can be found as “.csv “ files (“pheno_indica.csv” and “pheno_japonica”), marker positions can be found in files “map_indica.csv” and “map_japonica.csv”, and EC data are available in files “EC_indica.csv” and “EC_japonica.csv”. Supplemental material available at Figshare: https://doi.org/10.25387/g3.7685636.

## Results

### Phenotypic data analysis

The *indica* dataset was balanced with a total of 327 lines per environment, while the *tropical japonica* dataset was unbalanced, with a total of 23 lines common to all environments (Table 2). Estimations of broad-sense heritability estimated on a line-mean basis per trait by year for both datasets were medium to high, with PHR having the highest values of heritability in both datasets.

**Table 2:** Description of the rice breeding lines evaluated each year and broad-sense heritabilities for each trait calculated in a line-basis. GY: grain yield, PHR: percentage of head rice, GC: percentage of chalky grains, PH: plant height

*indica*					
		H^2^			
Year	Lines evaluated	GY	PHR	GC	PH
2010	327	0.46*^a^*	0.86*^a^*	0.73*^a^*	0.49*^a^*
2011	327	0.60*^a^*	0.78*^a^*	0.59*^a^*	0.66*^a^*
2012	327	0.68*^a^*	0.71*^a^*	0.69*^a^*	0.58*^a^*
*tropical japonica*					
2009	93	0.44	0.67	0.59	0.77
2010	292	0.68	0.71	0.59	0.62
2011	319	0.43*^a^*	0.85*^a^*	0.41*^a^*	0.62*^a^*
2012	319	0.57*^a^*	0.79*^a^*	0.75*^a^*	0.79*^a^*
2013	134	0.70	0.75	0.80	0.78

a Previously reported by Monteverde *et al.* (2018) and Rosas *et al.* (2017)

Table 3 shows the partitioning of the observed phenotypic variance into different sources of variation for both rice datasets. In the *indica* population, PHR and GC showed the highest proportion of variance explained by G×Y, at 20.04% and 13.22%, respectively. On the other hand, the year component was the highest variance component for GY and PH (Table 3). In the *tropical japonica* population, the year component was the highest; it was above all components for the four traits, and much higher than for the *indica* population. In contrast, the G×Y component was lower in *tropical japonica* compared to *indica* (Table 3).

**Table 3:** Trait variance component estimation and proportion of the total variance explained for the four traits evaluated in Uruguayan *indica* and *tropical japonica* populations. GY: grain yield, PHR: percentage of head rice, GC: percentage of chalky grains, PH: plant height

*indica*					
GY			PHR		
Group	Variance	%	Group	Variance	%
Year	496540	18.9	Year	0.0001	10.02
Genotype	379554	14.5	Genotype	0.0002	20.04
GxY	143374	5.5	GxY	0.0002	20.04
Column	55361	2.1	Column	0.000007	0.70
Row	31357	1.2	Row	0.000006	0.60
Block	516792	19.7	Block	0.0002	20.04
Residual	1000130	38.1	Residual	0.000285	28.56
GC			PH		
Group	Variance	%	Group	Variance	%
Year	0.0003	13.22	Year	12.51	9.26
Genotype	0.0004	17.62	Genotype	8.91	6.60
GxY	0.0003	13.22	GxY	5.56	4.12
Column	0.0003	13.22	Column	0.84	0.62
Row	0.00007	3.08	Row	0.92	0.68
Block	0.0005	22.03	Block	8.31	6.15
Residual	0.0004	17.62	Residual	98.02	72.57
*tropical japonica*							
GY			PHR		
Group	Variance	%	Group	Variance	%
Year	1988252	43.2	Year	0.001	41.36
Genotype	197961	13.2	Genotype	0.0003	12.41
GxY	99052	5.1	GxY	0.0001	4.14
Column	24932	0.3	Column	0.000008	0.33
Row	19062	0.5	Row	0.00001	0.41
Block	115775	22.7	Block	0.0006	24.81
Residual	682613	15.1	Residual	0.0004	16.54
GC			PH		
Group	Variance	%	Group	Variance	%
Year	0.005	66.32	Year	37.5	43.7
Genotype	0.0007	9.29	Genotype	18.4	21.4
GxY	0.0006	7.96	GxY	1.6	1.8
Column	0.000009	0.12	Column	0.1	0.1
Row	0.00003	0.40	Row	0.1	0.2
Block	0.0004	5.31	Block	13.3	15.5
Residual	0.0008	10.61	Residual	14.8	17.2

### Genomic prediction of untested years

**Figure 1:**
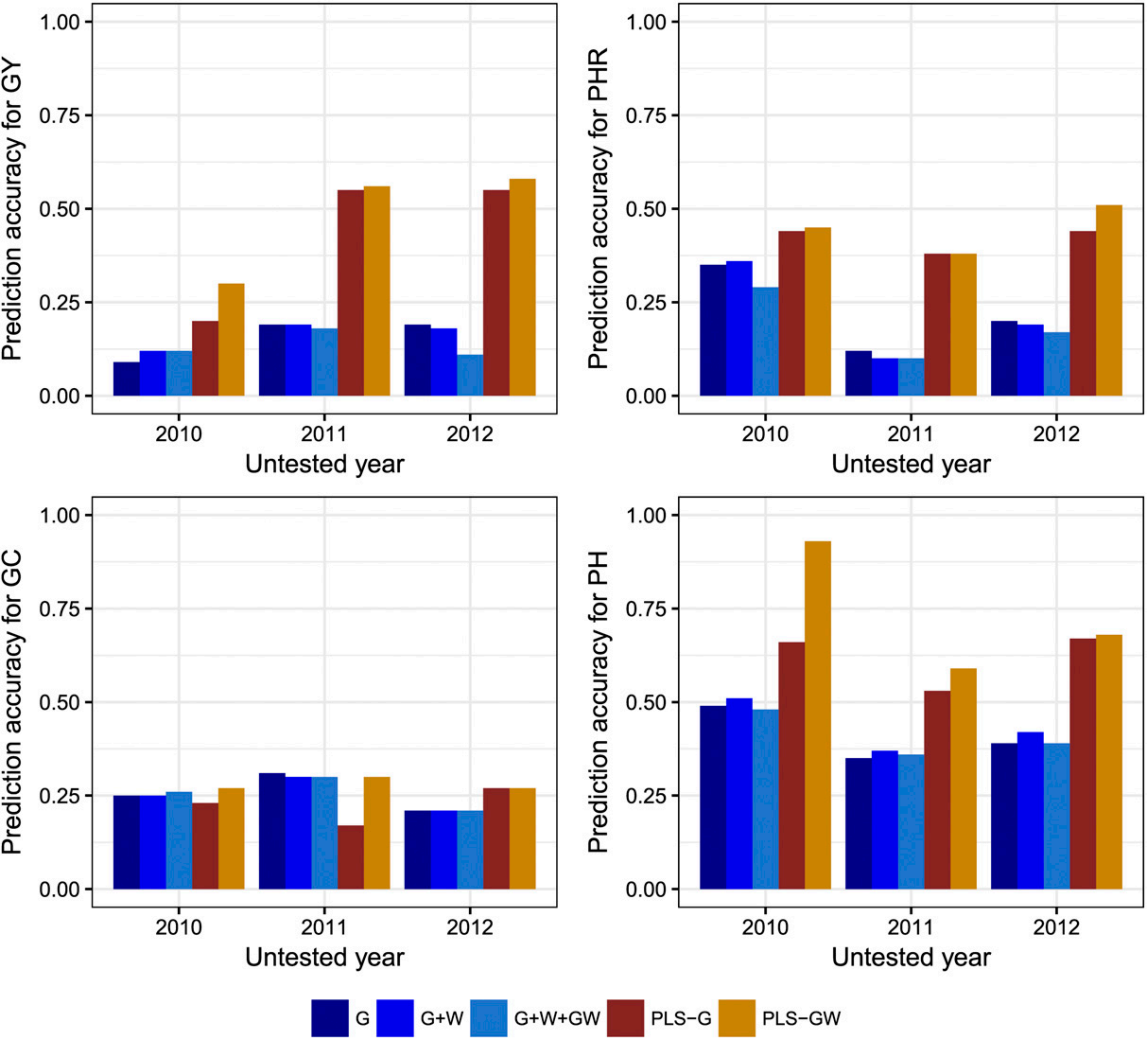
Correlations between predicted *vs.* observed values for the “leave one year out” prediction scenario for Grain Yield (GY), Head Rice Percentage (PHR), Grain Chalkiness percentage (GC) and Plant Height (PH) for predicting untested years with the G, G+W, G+W+GW, PLS-G and PLS-GW for the *indica* rice breeding population. G = genotypic main effect modeled with marker covariates, W = Environmental main effect modeled with EC, GW = interaction between genotypic and environmental effects, PLS-G = Partial least squares using marker covariates as predictors, PLS-GW = Partial least squares using marker covariates and EC as predictors.

Bar plots showing prediction accuracy for the four traits in the *indica* population are shown in Figure 1. PLS-based methods showed higher prediction accuracies than reaction norm-based models for all traits except GC, where prediction accuracies for the PLS method using both markers and EC (PLS-GW) were the same as the reaction norm models. For PLS models, the use of EC in addition to molecular markers resulted in higher prediction accuracies in all cases, though PHR in 2011 and GC in 2012 had identical prediction accuracies for both methods. For reaction norm models, fitting the main effect of genotypes, environments and interaction (G+W+GW model) resulted in either lower or equal prediction accuracies than fitting the simpler model without the interaction term (G+W model) (Figure 1).

**Figure 2:**
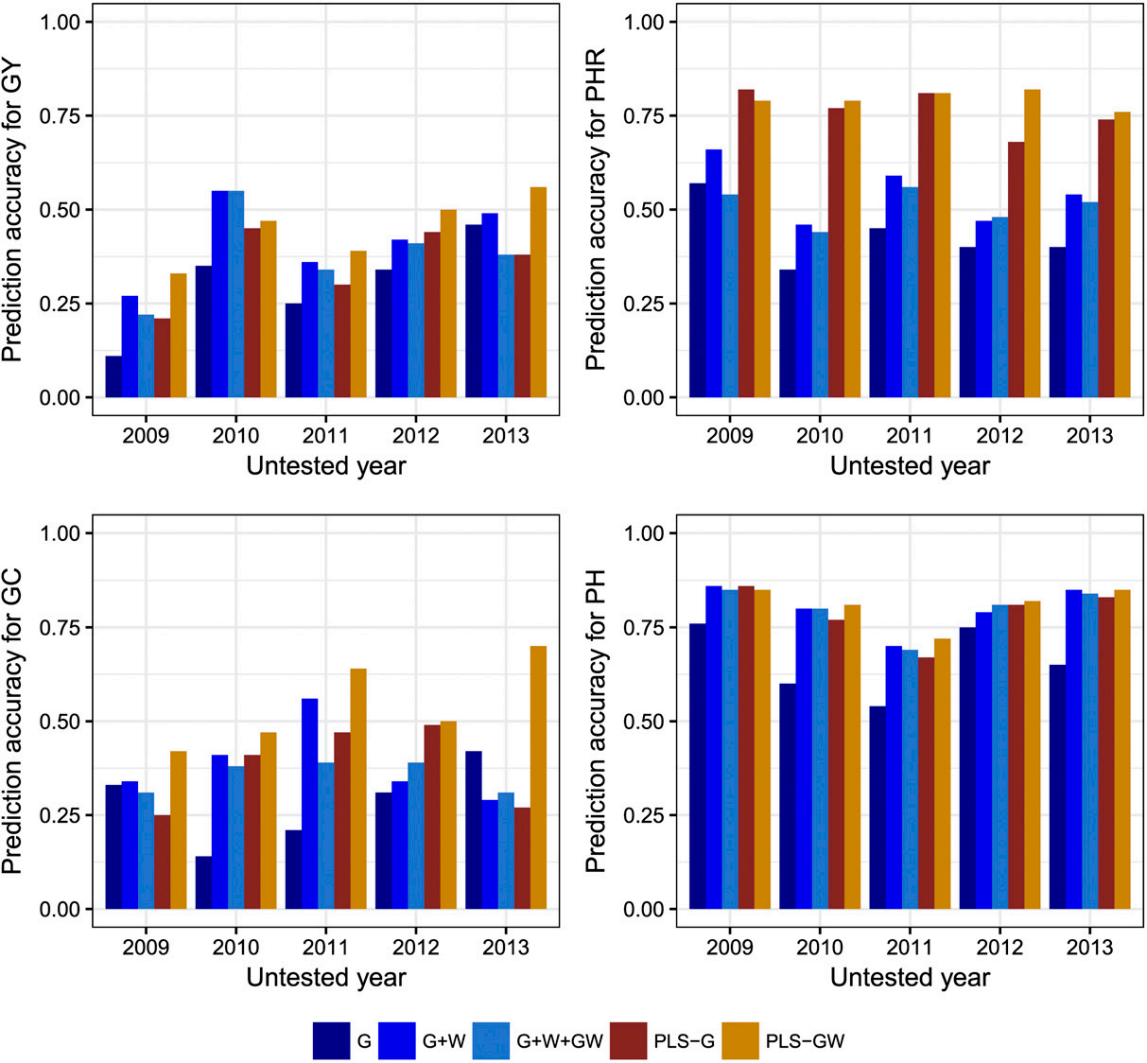
Correlations between predicted *vs.* observed values for the “leave one year out” prediction scenario for Grain Yield (GY), Head Rice Percentage (PHR), Grain Chalkiness percentage (GC) and Plant Height (PH) for predicting untested years with the G, G+W, G+W+GW, PLS-G and PLS-GW for the *tropical japonica* rice breeding population. G = genotypic main effect modeled with marker covariates, W = Environmental main effect modeled with EC, GW = interaction between genotypic and environmental effects, PLS-G = Partial least squares using marker covariates as predictors, PLS-GW = Partial least squares using marker covariates and EC as predictors.

In the *tropical japonica* population, the use of PLS-based models was always better than reaction norm models, with the single exception of GY in 2010 (Figure 2). In all cases, including both markers and EC (PLS-GW) was better than using markers only (PLS-G). Within the reaction norm models, the G+W method was the best, with the exception of GC in 2013. Fitting a G×E component in these models resulted in lower prediction accuracies than fitting the G+W model (Figure 2).

Many of the EC used in this study were correlated. This may result in lower prediction accuracies in the reaction norm model, since it would weight the environmental covariances toward the highly correlated variables. Reaction norm models with a subset of less correlated variables were tried, resulting in very similar or even lower prediction accuracies than when using the entire set of EC (Table S2).

When running PLS with all the environments within each dataset, we can detect which variables best explain each trait by looking at the coefficients. Table 4 shows the ranking of coefficients for the EC variables for each trait in both datasets. For GY, variables related to temperature and humidity during flowering stage were among the most important. For PHR and GC, the 5 variables with the highest coefficients were related to temperature, humidity and solar radiation during maturation. In the *tropical japonica* dataset, variables related to humidity, solar radiation and rainfall during maturation showed the highest coefficients for PHR and GC. For GY, two variables at flowering time showed higher coefficient values than the rest: maximum temperature and wind speed (Table 4).

**Table 4:** Top 5 PLS-GW coefficients for the environmental covariates for Grain Yield (GY), Head Rice Percentage (PHR), Grain Chalkiness percentage (GC) and Plant Height (PH) for the *indica* and *tropical japonica* rice breeding populations. Suffixes V, R and M mean Vegetative stage, Reproductive stage, and Maturation stage respectively

*indica*					
		Coefficient			
Type	Variable	GY	PHR	GC	PH
Temperature	ThermAmp_V	—	0.0001251	−0.000403	—
	MinTemp_R	0.005277	—	—	−0.00751
	MeanTemp_R	0.005265	—	—	−0.00773
	ThermAmp_M	—	0.0001273	−0.000402	—
Precipitation	EfPpit_R	0.005159	—	—	−0.00723
Evaporation	TankEv_V	0.005185	—	—	−0.00772
	TankEv_M	—	0.0001272	−0.000407	—
Humidity	MinRelH_M	—	−0.0001270	0.000406	—
Radiation	SolRad_M	—	0.0001267	−0.000402	—
Wind	Wind_V	−0.005108	—	—	0.00766
*tropical japonica*					
		Coefficient			
Type	Variable	GY	PHR	GC	PH
Temperature	MinTemp_V	—	—	—	−0.014
	MaxTemp_V	0.0102	—	—	−0.015
	MeanTemp_V	—	—	—	−0.016
	DegDayRice_V	—	—	—	−0.016
	MaxTemp_R	−0.0125	—	—	—
	AvTemp_M	−0.0101	—	—	—
Precipitation	PpitDay_R	—	—	—	0.014
	EfPpit_M	—	0.013	0.018	—
	AccumPpit_M	—	−0.013	—	—
Evaporation	PicheEv_V	—	0.013	—	—
Humidity	MinRelH_M	—	−0.015	0.019	—
Radiation	Sunhs_M	—	0.013	−0.018	—
	SolRad_M	—	—	−0.017	—
	RelSun_M	—	—	−0.017	—
Wind	Wind_V	−0.0103	—	—	—
	Wind_R	−0.0126	—	—	—

### Detecting QTL in single environments

We searched for significant trait-marker associations in single years to find QTL to test for interactions with EC in the next step. In this first analysis, we could not find any QTL that passed the FDR threshold for GY in any environment in any population. In the case of PH, we did not find any QTL for the *indica* population, but we found one major effect QTL on chromosome 1 (position: 37,755,448 - 38,755,448 bp) that was significant in all environments in the *japonica* dataset; it corresponds to the *sd-1* gene (position: 38,363,881 bp).

We detected QTL for grain quality traits in both datasets (Table 5). In the *indica* population, a total of 13 QTL (chromosomes 1, 2, 3, 4, 6, 7, 10 and 11) were found for PHR, and a total of 4 QTL (chromosomes 1, 3 and 4) for GC. QTL were found only in years 2010 and 2012 for PHR, and in years 2011 and 2012 for GC. Three of the QTL were reported in a previous GWAS analysis using this same dataset (Quero *et al.* 2018). These QTL were: qPHR.i.2.2 (S2_24210614), qPHR.i.3.1 (S3_10247958), qGC.i.1.1 (S1_1066894). Two additional QTL were in LD with two previously reported QTL in the same study. These were qPHR.i.3.2 and qPHR.i.6.1, which were in LD with S3_15365726 and S6_829223 in our study, respectively. In the *tropical japonica* population, a total of 5 QTL were found for PHR (chromosomes 1, 2, 3, 6, and 8), and one for GC (chromosome 6) (Table 5). Two of these QTL were in LD with previously reported QTL: qPHR.j.3.1 with S3_1395165, and qGC.j.6.2 with S6_27402260 (Quero *et al.* 2018). No significant QTL were found for GY or PH for any year in either of the populations.

**Table 5:** Marker-trait associations for percentage of head rice (PHR) and percentage of chalky grain (GC) traits in *indica* and *tropical japonica* rice breeding populations. Chromosome position (bp), year, effect of the alternative allele, and score (-log_10_(p-value)) are shown in the table

*indica*					
Marker	Chr	Position	Year	Alt allele effect (%)	Score
PHR					
S1_1015065	1	1015065	2010	−1.62	4.98
S2_24210614	2	24210614	2012	2.95	7.20
S3_8880979	3	8880979	2010	1.32	4.60
S3_10247958	3	10247958	2010	−2.12	9.97
S3_15365726	3	15365726	2010	−1.85	7.19
S4_29728982	4	29728982	2010	1.30	4.68
			2012	2.37	5.66
S6_829223	6	829223	2010	1.76	5.04
S6_11022101	6	11022101	2012	−2.10	5.30
S6_13215923	6	13215923	2010	−1.24	5.47
S6_21327503	6	21327503	2012	2.63	5.54
S7_14798606	7	14798606	2010	1.25	5.05
S10_6737554	10	6737554	2010	2.40	5.64
S11_24425810	11	24425810	2010	1.92	4.70
GC					
S1_1066894	1	1066894	2011	1.33	4.06
S1_22492066	1	22492066	2012	1.45	5.74
S3_16037360	3	16037360	2011	0.72	4.07
S4_22480721	4	22480721	2011	1.24	4.14
*tropical japonica*					
PHR					
S1_38686312	1	38686312	2013	2.0	3.20
S2_27660046	2	27660046	2013	−1.0	4.41
S3_1395165	3	1395165	2011	1.0	6.18
S6_27834772	6	27834772	2011	−2.0	4.89
			2013	−1.0	3.44
S8_23380395	8	23380395	2013	−2.0	4.11
GC					
S6_27402260	6	27402260	2011	2.00	4.62
			2013	2.00	3.94

### QTL × environmental covariate interactions

A decomposition of the QTL with significant QTL × environment interaction was obtained by introducing environmental covariates as explanatory variables. We first tested different covariance structures for the modeling of the G×E component and compared them using BIC (see Methods). For all QTL and traits, BIC values decreased when using the HCS matrix compared to the CS matrix. However, the HCS model already behaves quite similar to the maximally complex UN model, so the HCS was the model of choice (Table S1). The QTL responses for the *indica* dataset are shown in Table 6. One QTL showed significant interaction with environmental covariates related to precipitation and humidity during the maturation stage. Marker S2_24210614 showed a negative relationship with PpitDay_M and RelH_M. The high correlation between these two variables (*r* = 0.99) explains why they show the same coefficients for the main QTL effect (α), and the interaction (β). No significant main effect was detected for this QTL (Table 6).

**Table 6:** QTL responses to EC for percentage of head rice (PHR) in the *indica* rice population. Suffixes R and M mean Reproductive stage, and Maturation stage respectively

Trait	Marker	Chromosome	Position	EC	α	β
PHR	S2_24210614	2	24210614	PpitDay_M	0.09	−0.03*
				RelH_M	0.08	−0.04*
				MinTemp_R	0.10	−0.04*

α: QTL main effect.

β: Slope parameter for the QTL×EC parameter.

* significance level at α = 0.05.

Results for regression of marker covariates on environmental covariates for the *tropical japonica* dataset are shown in Table 7. For GC, marker S6_27402260, located in chromosome 6, showed a significant positive response to weather covariates related to precipitation and minimum temperature, and a negative response to sunshine duration and solar radiation. This marker also showed a significant main effect (Table 7).

**Table 7:** QTL responses to EC for head rice percentage (PHR) and percentage of chalky grain (GC) in the *tropical japonica* rice population. Suffixes R and M mean Reproductive stage, and Maturation stage respectively

Trait	Marker	Chromosome	Position	EC	α	β
GC	S6_27402260	6	27402260	TempMin15_M	0.2*	−0.08*
				MinTemp_M	0.3*;	0.3*
				PpitDay_M	0.4*	0.07*
				SolRad_M	0.3*	−0.2*
				RelSun_M	0.4*	−0.08*
				Sunhs_M	0.4*	−0.7*

α: QTL main effect.

β: Slope parameter for the QTL×EC parameter.

* Significance level at α = 0.05.

## Discussion

In this work we proposed to characterize and interpret G×E interaction for four traits (GY, PHR, GC and PH) in two different breeding populations of rice (*indica* and *tropical japonica*) grown in a subtropical/temperate climate. In the first part of our paper, we compare the performance of different genomic prediction models that account for genotype, environment and G×E components, to predict untested years, and we identify the most influential weather covariates for our two datasets. In the second part, we map environment-specific QTL and study the environmental variables that affect their expression, in order to interpret the QTL×E effects that account for the total G×E.

### Prediction accuracies for untested environments

Usually genomic prediction models are tested and compared using cross-validation strategies. In a multiple environment context, most studies include two basic random cross-validation schemes (Burgueño *et al.* 2012): CV1, which tests the performance of lines that have not been evaluated in any of the observed environments, and CV2, which tests the performance of lines that have been evaluated in some environments but not in others. These two scenarios have the disadvantage of training and validating the models with the same data, which could lead to an overestimation of the prediction accuracy the model would attain if it had been applied in an independent test dataset. Predicting new environments is a more difficult task but could represent a good validation strategy because the performance of prediction models is assessed in an independent dataset. In this work we used a cross-validation scheme for prediction in untested environments, represented by years, a component of G×E that is not easy to reproduce. This is a very relevant type of prediction for a small plant breeding program, where data from multiple locations is either limited or absent, and the need is to predict which lines are more likely to perform better in future environments. The use of EC to model the environment component explicitly has been previously shown to increase prediction accuracies for untested environments (Malosetti *et al.* 2016, Jarquín *et al.* 2017), and this situation also applies to our work.

For prediction, we compared two modeling approaches that differ in the way that multiple and correlated variables are handled: 1) a variance components approach that allows modeling the main and interaction effects of markers and EC using covariance structures, and 2) a PLS approach that models genotype and environment effects by identifying a linear combination of all the explanatory variables, providing latent vectors that optimally predict the response variable. We found that the PLS-GW model was in all cases superior to or not different from PLS-G and reaction norm models in both datasets. Although the variance explained by the G×E component in the *indica* population, shown in Table 3, was comparable in some cases to the variance explained by the genotype and/or the year main components, the proportion of variance explained jointly by the genotype, environment and G×E components, was never superior to 50% of the total variance. This could explain the lower prediction accuracies obtained in this population compared to the *japonica* population. It is possible that the EC used in this study explained only a limited proportion of the across environment interaction in the *indica* dataset, and for this reason reaction norm models, when fitting covariance matrices for the environment and marker by environment interaction, did not improve prediction accuracies in comparison to the simpler GBLUP model. In the *japonica* population, the proportion of the total variance explained by G×E was very low compared to the main genotype and environment components, which also explains why modeling a specific interaction covariance matrix did not give better results than modeling the main genotype and environment covariance matrices alone. In this population, the main environment effect was better represented by the EC, and thus, prediction accuracies, when including an EC covariance matrix (W) or the EC in the PLS model, were higher than when using a G matrix or molecular markers alone.

Besides the ability of handling numerous and correlated predictors, an additional advantage of using PLS models is that we can detect which covariates are the most explanatory in our model by looking at the model coefficients (Wold *et al.* 2001; Mehmood *et al.* 2012). Previous studies have shown the benefits of PLS for identifying the set of EC that best explain G×E (Vargas *et al.* 1998; Vargas *et al.* 1999; Crossa *et al.* 1999). In these studies, the G×E component of the trait was used as a response and regressed to EC only. In our case, we decided to report the results of the regression of the trait means to both EC and markers, since regressing the G×E component to EC resulted in increased MSEP with an increasing number of components, and thus a poor model fit. For GY, minimum and average temperature, and effective precipitation during flowering time showed the highest positive coefficients for *indica* rice, as shown in Table 4. In regions with a temperate climate, low temperatures during flowering can affect grain yield by inducing spikelet sterility (Yoshida 1981, Alvarado 2002). The probability of occurrence of temperatures under 15° during January (when rice usually enters the flowering stage) in Eastern Uruguay is about 20%, and would be most detrimental for *indica* varieties, which are best adapted to tropical climates. In the *tropical japonica* population, the two EC that showed the highest (negative) coefficients for GY were wind speed during flowering, and maximum temperature during grain filling. Both wind speed and high temperatures during reproduction have been proven to negatively affect GY due to pollen dehydration and consequent spikelet sterility (Marchezan and Aude 1993; Raju *et al.* 2013).

For the grain quality traits, EC related to humidity, solar radiation and sunshine duration during grain ripening were among the most important in both datasets. The positive coefficients for solar radiation, and the negative coefficients for humidity reflect the relative effects of these variables on milling quality, as previously reported (Siebenmorgen *et al.* 2012; Edwards *et al.* 2017). Many studies have reported negative effects of high temperatures on grain chalk and percent head rice (Tashiro and Wardlaw, 1991, Lyman *et al.* 2013). For example, for *japonica* cultivars, temperatures higher than 26° Can cause chalky grain appearance (Chen *et al.* 2016), but maximum daytime temperatures higher than 33° Cause dramatic changes in the distribution of head and broken rice, and increase the proportion of chalky grain (Ambardekar *et al.* 2011; Lyman *et al.* 2013). In Eastern Uruguay, maximum temperatures during February-March, the period in which rice kernels usually develop, rarely reach 32°. In our own dataset, the average maximum temperatures were never higher than 30°, so it is probable that in the absence of high stress-inducing temperatures in sub-tropical rice growing areas, other variables such as humidity and solar radiation are more important, as is reflected in our results.

### QTL detection and interaction With environmental covariates

For this part of the analysis we used mixed-models to analyze QTL by EC interactions because of their flexibility, and the possibility of modeling genetic correlations between environments. We first performed an association mapping analysis for each of the four traits in each environment in both populations. In the case of PH, Rosas *et al.* (2017) performed a GWAS analysis on these same populations using the mean across environments and found a major effect QTL corresponding to the *sd-1* gene which was segregating in the *japonica* population, but fixed in the semi-dwarf *indica* population (Rosas *et al.* 2017). When we performed a single environment scan we could not find any other QTL in either population, other than a major-effect QTL corresponding to the *sd-1* gene in *japonica*.

Of the 23 QTL we found for PHR and GC in both populations in Table 5, 8 were coincident with QTL reported by Quero *et al.* (2018) in the same populations using the mean across environments. For PHR in *indica*, we found evidence of one genomic region, located in chromosome 2 that is affected by humidity, one of the main environmental factors that affect milling quality in rice (Table 6) (Cooper *et al.* 2008, Zhao and Fitzgerald 2013).

Two putative QTL in *tropical japonica* were co-located on chromosome 6: S6_27834772 for PHR and S6_27402260 for GC. These two QTL are in LD with qPHR.j.6.1 and qGC.j.6.2 previously found by Quero *et al.* (2018), and contain genes related to starch metabolism, such as *OsBEI* (LOC_Os06g51084). It is known that the expression of starch branching enzymes, like *OsBEI*, can be affected by temperature (Yamakawa *et al.* 2007; Sreenivasulu *et al.* 2015). According to our results, QTL S6_27402260 showed interaction with low temperature, precipitation, sunshine duration and solar radiation for GC, as shown in Table 7. Other researchers have shown that periods of intense solar radiation and high humidity during the ripening stage can increase the incidence of chalky grains (Wakamatsu *et al.* 2009, Zhao *et al.* 2016). But these reports do not constitute proof that there is a causal relationship between the expression of these QTL and the EC, because many EC are correlated in a complex way and not all EC were observed. In temperate climates, where day and night temperatures are never as high as in the tropics, other environmental factors such as humidity and solar radiation can affect milling quality in a negative way. These findings should be confirmed by analyzing more lines in more environments to properly quantify QTL main and environment-specific effects.

The approach of mapping QTL by environment interaction used in this study requires a QTL to have a strong effect in a specific environment. This poses the limitation that QTL with smaller effects in individual environments but capable to explain larger proportions of the observed phenotypic G×E may be overlooked. In our datasets, the proportion of phenotypic variance explained by the G×Y component is low in the *tropical japonica* dataset (1.8–7.96%, Table 3), but higher in the *indica* dataset (4.12–20%). However, approaches for testing for genotype by year interaction at each SNP were performed and no significant QTL were found.

In this work we used PLS, multiplicative reaction norm and mixed models to analyze our data, predict genotypic performance for yield, height and milling quality traits, and detect QTL by EC interactions. In all these analyses we assumed that the relationships between molecular markers and EC were linear, which constitutes a major limitation since interactions between genes and environmental conditions may take many different forms. A next step would be to fit statistical models with more biological realism, using models that could accommodate non-linear and more complex responses over a more extensive number of environments. Crop growth models also hold promise as a way to integrate more complex biological knowledge into the prediction process of G×E (Malosetti *et al.* 2016). Although rather small, our two datasets allowed us to extract some broad conclusions about the nature of G×E in the Uruguayan mega-environment. Additional research, including more environments and modeling non-linear relationships between genes and EC, will be of particular value to better understand and predict the nature of G×E for commercially relevant traits of rice grown in temperate regions. Results from PLS and QTL by EC interactions suggest that in temperate and subtropical regions, humidity and solar radiation may have a stronger influence on milling quality traits than temperature, due to the fact that temperatures in these regions are never as high as in the tropics.
